# Predictive and Prognostic Assessment Models for Tumor Deposit in Colorectal Cancer Patients With No Distant Metastasis

**DOI:** 10.3389/fonc.2022.809277

**Published:** 2022-02-16

**Authors:** Jingyu Chen, Zizhen Zhang, Jiaojiao Ni, Jiawei Sun, Wenhao Ren, Yan Shen, Liuhong Shi, Meng Xue

**Affiliations:** ^1^Department of Gastroenterology, The Second Affiliated Hospital of Zhejiang University School of Medicine, Hangzhou, China; ^2^Institute of Gastroenterology, Zhejiang University, Hangzhou, China; ^3^Department of Gastrointestinal Oncology, Peking University Cancer Hospital and Institute, Beijing, China; ^4^Shulan International Medical College, Zhejiang Shuren University, Hangzhou, China; ^5^Department of Pathology, Peking University Cancer Hospital and Institute, Beijing, China; ^6^School of Medicine, Ningbo University, Ningbo, Zhejiang, China; ^7^Department of Ultrasound, The Second Affiliated Hospital of Zhejiang University School of Medicine, Hangzhou, China

**Keywords:** tumor deposit, colorectal cancer, nomogram, SEER program, survival

## Abstract

**Background:**

More and more evidence indicated that tumor deposit (TD) was significantly associated with local recurrence, distant metastasis (DM), and poor prognosis for patients with colorectal cancer (CRC). This study aims to explore the main clinical risk factors for the presence of TD in CRC patients with no DM (CRC-NDM) and the prognostic factors for TD-positive patients after surgery.

**Methods:**

The data of patients with CRC-NDM between 2010 and 2017 were extracted from the Surveillance, Epidemiology, and End Results (SEER) database. A logistic regression model was used to identify risk factors for TD presence. Fine and Gray’s competing-risk model was performed to analyze prognostic factors for TD-positive CRC-NDM patients. A predictive nomogram was constructed using the multivariate logistic regression model. The concordance index (C-index), the area under the receiver operating characteristic (ROC) curve (AUC), and the calibration were used to evaluate the predictive nomogram. Also, a prognostic nomogram was built based on multivariate competing-risk regression. C-index, the calibration, and decision-curve analysis (DCA) were performed to validate the prognostic model.

**Results:**

The predictive nomogram to predict the presence of TD had a C-index of 0.785 and AUC of 0.787 and 0.782 in the training and validation sets, respectively. From the competing-risk analysis, chemotherapy (subdistribution hazard ratio (SHR) = 0.542, *p* < 0.001) can significantly reduce CRC-specific death (CCSD). The prognostic nomogram for the outcome prediction in postoperative CRC-NDM patients with TD had a C-index of 0.727. The 5-year survival of CCSD was 17.16%, 36.20%, and 63.19% in low-, medium-, and high-risk subgroups, respectively (Gray’s test, *p* < 0.001).

**Conclusions:**

We constructed an easily predictive nomogram in identifying the high-risk TD-positive CRC-NDM patients. Besides, a prognostic nomogram was built to help clinicians identify poor-outcome individuals in postoperative CRC-NDM patients with TD. For the high-risk or medium-risk subgroup, additional chemotherapy may be more advantageous for the TD-positive patients rather than radiotherapy.

## Introduction

Colorectal cancer (CRC), the most commonly diagnosed gastrointestinal malignant tumor and the second leading cause of cancer-related deaths worldwide, is a heavy health burden nowadays (1). Thanks to universal screening and novel therapeutic strategies for advanced disease, the overall incidence and mortality of CRC have declined over the past 30 years (2). The tumor stage at diagnosis is the most powerful predictor of survival. The 5-year relative survival rate for CRC ranges from 90% for stage II to 14% for stage IV. Encouragingly, CRC patients with no distant metastasis (CRC-NDM) still have a relatively good prognosis after comprehensive management, including surgery resection, systemic adjuvant therapy, and immunotherapy (3).

Tumor deposit (TD) in CRC, defined as a discrete nodule of tumor in the pericolonic and perirectal adipose tissue or adjacent mesentery without identifiable vascular structure or lymph node, has been the controversial point for many years (4). Over the past decades, increasing evidence suggested modifications of TD in the TNM staging system. For the fifth and sixth editions, the TD was included in the T or N category according to the size or contour of TD (5). As a kind of invasion and metastasis, TD has even been advised by some investigators to be classified into the M category (6, 7). However, TD was only categorized into the N1c category in CRC patients without lymph node metastasis (LNM) since the seventh TNM staging system in 2009 (8).

There is accumulating evidence that the prognostic implications and role of TD are not sufficiently recognized in current staging systems (9). Several studies revealed that the presence of TD in the resected specimen was an independent and powerful prognostic factor for CRC patients after surgery, regardless of the lymph node status (10–12). What is more, many studies also indicated that TD counting was independently associated with poor outcomes and proposed to add TD to the number of LNM (13–15). A meta-analysis incorporating 17 retrospective studies found that TD presence was a stronger predictive factor than LNM or extramural vascular invasion (EMVI) for liver, lung, and peritoneal metastases (16). In addition, TD presence was also significantly associated with higher local recurrence and poorer outcomes for patients with rectal adenocarcinoma (17, 18).

For the diagnosis of TD, a recent retrospective study indicated that preoperative MRI with the incorporation of texture analysis parameters, morphological parameters, and lesion shape was helpful in differentiating TD from LNM. However, this kind of MRI can only recognize TD with size > 1 cm and EMVI (19), and the availability limited their usage in clinical practice. At present, pathology is still the main way to diagnose TD. Therefore, a high-quality review of the surgical field and resection specimens is essential for the diagnosis of TD, especially for small TD.

However, the clinical risk factors for TD presence in patients with CRC-NDM and the prognostic factors for postoperative CRC-NDM patients with TD are both poorly explored. For TD-positive patients, the effect of chemotherapy and radiotherapy also remains rarely reported. In the present study, we analyzed the data from the Surveillance, Epidemiology, and End Results (SEER) database and tried to clarify these factors. A predictive nomogram was built to predict the probability of TD presence by incorporating independent risk factors for TD in our study, which may be an easy tool to assist surgeons or pathologists in identifying high-risk TD. What is more, Fine and Gray’s competing-risk model was used to analyze independent prognostic factors for CRC-specific death (CCSD) in postoperative CRC-NDM patients with TD. Besides, a competing-risk nomogram was also developed for clinicians to assess whether these patients of unfavorable outcome merit further adjuvant therapy at all.

## Methods

### Patients

The data of patients with CRC-NDM were obtained from the National Cancer Institute’s SEER Cancer database released in April 2021 with a private ID (11505-Nov2020). The treatment data were collected from SEER plus data *via* another application.

The inclusion criteria of eligible patients were as follows: a) CRC-NDM patients aged over 18 years were diagnosed between January 2010 and December 2017; b) patients received radical surgical resection; c) patients were pathologically diagnosed, and CRC was the only primary cancer; d) records of lymph node and TD status were available; and e) records of survival data (including cause-specific death classification and survival months) were clear. Patients with unknown information of detailed age at diagnosis, gender, race, T stage, tumor size, differentiated grade, radiotherapy sequence, and follow-up were excluded.

### Variables

Race was grouped into white, black, or other. Age was classified into ≤60 and >60 years. Gender was grouped into male or female. Primary site of the tumor was divided into four parts: right colon (from the cecum to the transverse colon), left colon (from the splenic flexure to rectosigmoid junction), rectum, and overlapping/not otherwise specified (NOS). Grade was grouped as well, moderately, poorly differentiated, and undifferentiated. Tumor size was regrouped into 4 groups: ≤2 cm, ≤3 cm (2 cm < tumor size ≤ 3 cm), ≤5 cm (3 cm < tumor size ≤ 5 cm), and >5 cm. Histology information was classified into adenocarcinoma (ICD-O-3 code include 8140/3, 8211/3, 8213/3, 8210/3, 8260/3, 8262/3, 8263/3, 8261/3, 8221/3), mucinous adenocarcinoma (ICD-O-3 code include 8480/3, 8481/3), and signet ring cell carcinoma (ICD-O-3 code include 8490/3). Number of positive lymph nodes (nLN) was divided into 0, 1–3, 4–6, and >7 according to the 8th American Joint Committee on Cancer (AJCC) N stage. Carcinoembryonic antigen (CEA) was classified into positive, negative, and unknown. Radiotherapy was regrouped into “no/unknown”, “before surgery”, “after surgery”, and “before and after surgery” according to the variable of radiation sequence. According to the SEER program, chemotherapy information was grouped as “yes” or “no/unknown”.

### Statistical Analysis

In the present study, both univariate and multivariate logistic regressions were performed to identify risk factors for TD. The odds ratio (OR) of variables was estimated and presented with 95% CIs. A predictive nomogram was built based on the results of the multivariate logistic regression model, and the C-index, area under the ROC curve (AUC), and calibration were used to evaluate its performance. The fit of the model was assessed by the Hosmer–Lemeshow test. All included patients were randomly grouped into the training set (60%) and validation set (40%) to develop and validate the predictive model.

For survival analysis of the postoperative CRC-NDM patients with TD presence, univariate and multivariate competing-risk models were utilized to analyze independent prognosis using Fine and Gray’s test (20). Patients with follow-up time of less than 3 months were excluded to avoid immortal time bias due to surgery-associated death. CCSD, defined as the time from diagnosis to death due to CRC, was the primary endpoint. Other causes of death were assumed as competing events. Cumulative incidence function (CIF) was performed to calculate the probability of CCSD among the categorical variables over time, and corresponding CIF curves were plotted at the same time. The subdistribution hazard ratio (SHR) for CCSD was calculated and presented with 95% CI. A competing-risk nomogram was built based on the result of the multivariate competing-risk model. The performance of the nomogram was evaluated in terms of the concordance index (C-index), calibration, and decision-curve analysis (DCA). Patients were further divided into three groups according to quartiles of predicted risk. All statistical analyses were conducted using R (version 3.6.3). A two-sided *p*-value was calculated, and statistical significance is declared for *p*-value <0.05.

## Results

### Patient Clinical Information

A total of 100,774 eligible cases were finally included in the present analysis. In this population, 10,735 (10.65%) had TD, and the remaining 90,039 (89.35%) did not. From a longitudinal point of data, TD-positive patients were younger and more frequently male and white. Besides, the primary lesion is often located in the right colon, presents in T3/T4 patients, and tends to have a larger tumor size. The histology was more often adenocarcinoma. The lymph node status was more often positive. **Table 1** depicts the detailed baseline characteristics of all patients in our study.

**Table 1:** Baseline clinical characteristics of patients in our study.

Variable	TD-negative		TD-positive	
	(n = 90,039)	%	(n = 10,735)	%
Age at diagnosis				
≤60	31,907	35.44	6,488	60.44
>60	58,132	64.56	4,247	39.56
Gender				
Female	44,357	49.26	5,194	48.38
Male	45,682	50.74	5,541	51.62
Race				
White	70,879	78.72	8,328	77.58
Black	10,149	11.27	1,195	11.13
Other	9,011	10.01	1,212	11.29
Primary site				
Right colon	45,300	50.31	4,662	43.43
Left colon	30,239	33.58	4,133	38.50
Rectum	13,291	14.76	1,761	16.40
Overlapping/NOS	1,209	1.34	179	1.67
Histology				
Adenocarcinoma	82,841	92.01	9,620	89.61
Mucinous Adenocarcinoma	6,649	7.38	891	8.30
Signet ring cell carcinoma	549	0.61	224	2.09
Grade				
Well differentiated	7,703	8.56	471	4.39
Moderately differentiated	68,347	75.91	7,212	67.18
Poorly differentiated	11,698	12.99	2,448	22.80
Undifferentiated	2,291	2.54	604	5.63
Tumor size				
≤2 cm	14,261	15.84	641	5.97
≤3 cm	14,099	15.66	1,458	13.58
≤5 cm	31,949	35.48	4,236	39.46
>5 cm	29,730	33.02	4,400	40.99
T stage				
T1	11,361	12.62	137	1.28
T2	16,629	18.47	504	4.69
T3	50,977	56.62	6,782	63.18
T4	11,072	12.30	3,312	30.85
nLN				
0	61,096	67.86	2,982	27.78
1–3	20,074	22.29	3,679	34.27
4–6	5,211	5.79	1,901	17.71
>7	3,658	4.06	2,173	20.24
CEA				
Positive	19,431	21.58	3,358	31.28
Negative	35,927	39.90	3,513	32.72
Unknown	34,681	38.52	3,864	35.99

≤3 cm, 2 cm < tumor size ≤ 3 cm; ≤5 cm, 3 cm < tumor size ≤ 5 cm.

NOS, not otherwise specified; TD, tumor deposit; nLN, number of positive lymph nodes; CEA, carcinoma embryonic antigen.

### Risk Factor Analysis of Tumor Deposit in Patients With Colorectal Cancer With No Distant Metastasis

**Figure 1:**
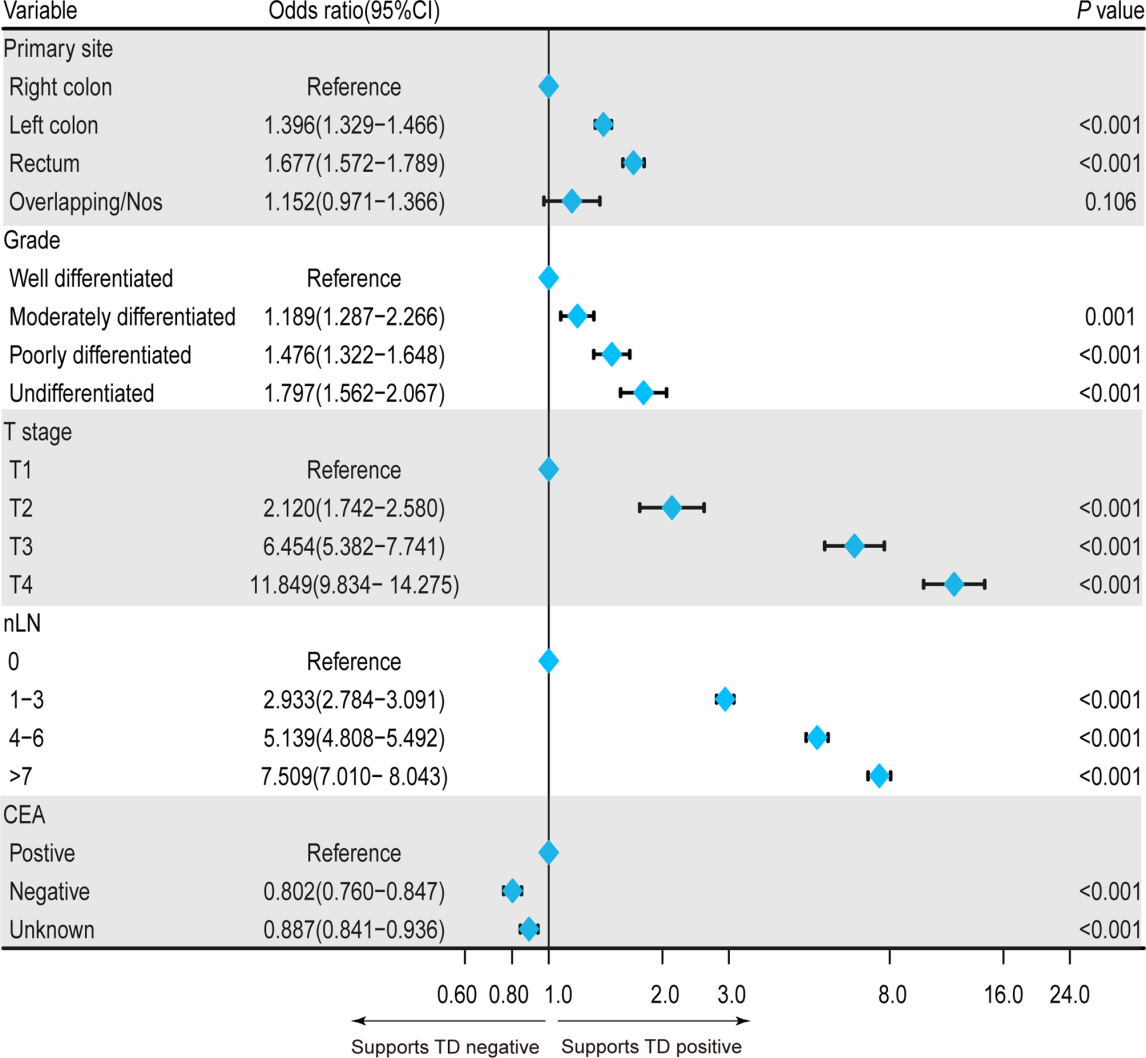
Forest plot showing the independent clinical risk factors for TD presence in patients with CRC-NDM. TD, tumor deposit; CRC-NDM, colorectal cancer with no distant metastasis.

To clarify the clinical risk factors of TD presence in patients with CRC-NDM, we employed a univariate logistic regression model to select significant candidate factors for TD, and a further multivariate logistic regression model was used to adjust confounding factors. As shown in **Table 2**, the result of the univariate model showed that race, age, histology, primary site, grade, tumor size, T stage, nLN, and CEA were significantly (*p* < 0.05) associated with TD. Then, these significant variables were selected to adjust for potential confounding factors by the multivariate regression model. In the multivariate model, poorer differentiated grade (*p* < 0.001), higher T stage (*p* < 0.001), positive CEA (*p* < 0.001), and more nLN (*p* < 0.001) were significantly associated with a higher risk of TD presence. As far as concerns the primary site, the left colon (*p* < 0.001) and rectum (*p* < 0.001) were inclined to have a higher risk of TD presence than the right colon, while overlapping/NOS (*p* = 0.106) had a comparable risk of TD presence as compared with the right colon (**Figure 1**).

**Table 2:** Univariate and multivariate logistic regression analyses to identify risk factors for the TD presence in patients with CRC-NDM.

Variable	Univariate analysis		Multivariate analysis	
	OR (95% CI)	*p*-Value	OR (95% CI)	*p*-Value
Age at diagnosis				
>60	Ref	―	Ref	―
≤60	1.193 (1.145–1.243)	**<0.001**	1.013 (0.968–1.059)	0.589
Gender			*NI*	
Female	Ref	―		
Male	1.036 (0.995–1.078)	0.085		
Race				
White	Ref	―	Ref	―
Black	1.002 (0.940–1.068)	0.948	1.001 (0.935–1.072)	0.974
Other	1.145 (1.074–1.221)	**<0.001**	1.025 (0.957–1.098)	0.478
Primary site				
Right colon	Ref	―	Ref	―
Left colon	1.328 (1.270–1.388)	**<0.001**	1.396 (1.329–1.466)	**<0.001**
Rectum	1.287 (1.215–1.365)	**<0.001**	1.677 (1.572–1.789)	**<0.001**
Overlapping/NOS	1.439 (1.226–1.688)	**<0.001**	1.152 (0.971–1.366)	0.106
Histology				
Adenocarcinoma	Ref	―	Ref	―
Mucinous Adenocarcinoma	1.154 (1.072–1.241)	**<0.001**	0.933 (0.869–1.017)	0.124
Signet ring cell carcinoma	3.514 (3.004–4.110)	**<0.001**	1.177 (0.987–1.403)	0.059
Grade				
Well differentiated	Ref	―	Ref	―
Moderately differentiated	1.726 (1.568–1.900)	**<0.001**	1.191 (1.076–1.317)	**0.001**
Poorly differentiated	3.422 (3.088–3.793)	**<0.001**	1.469 (1.316–1.640)	**<0.001**
Undifferentiated	4.312 (3.789–4.906)	**<0.001**	1.783 (1.550–2.051)	**<0.001**
Tumor size				
≤2 cm	Ref	―	Ref	―
≤3 cm	2.301 (2.091–2.532)	**<0.001**	1.037 (0.937–1.158)	0.503
≤5 cm	2.950 (2.708–3.213)	**<0.001**	0.996 (0.908–1.102)	0.943
>5 cm	3.293 (3.024–3.586)	**<0.001**	0.938 (0.855–1.040)	0.200
T stage				
T1	Ref	―	Ref	―
T2	2.513 (2.078–3.040)	**<0.001**	2.120 (1.742–2.580)	**<0.001**
T3	11.033 (9.305–13.081)	**<0.001**	6.454 (5.382–7.741)	**<0.001**
T4	24.806 (20.868–29.487)	**0.003**	11.849 (9.834–14.275)	**<0.001**
nLN				
0	Ref	―	Ref	―
1–3	3.755 (3.569–3.951)	**<0.001**	2.933 (2.784–3.091)	**<0.001**
4–6	7.474 (7.010–7.969)	**<0.001**	5.139 (4.808–5.492)	**<0.001**
>7	12.171 (11.410–12.983)	**<0.001**	7.509 (7.010–8.043)	**<0.001**
CEA				
Positive	Ref	―	Ref	―
Negative	0.566 (0.538–0.595)	**<0.001**	0.802 (0.760–0.847)	**<0.001**
Unknown	0.645 (0.614–0.677)	**<0.001**	0.887 (0.841–0.936)	**<0.001**

TD, tumor deposit; CRC-NDM, colorectal cancer with no distant metastasis; OR, odds ratio; NOS, not otherwise specified; nLN, number of positive lymph nodes; CEA, carcinoembryonic antigen. p-Value < 0.05 is displayed in bold.

### Construction and Validation of a Nomogram for Tumor Deposit Presence Probability Prediction

**Figure 2:**
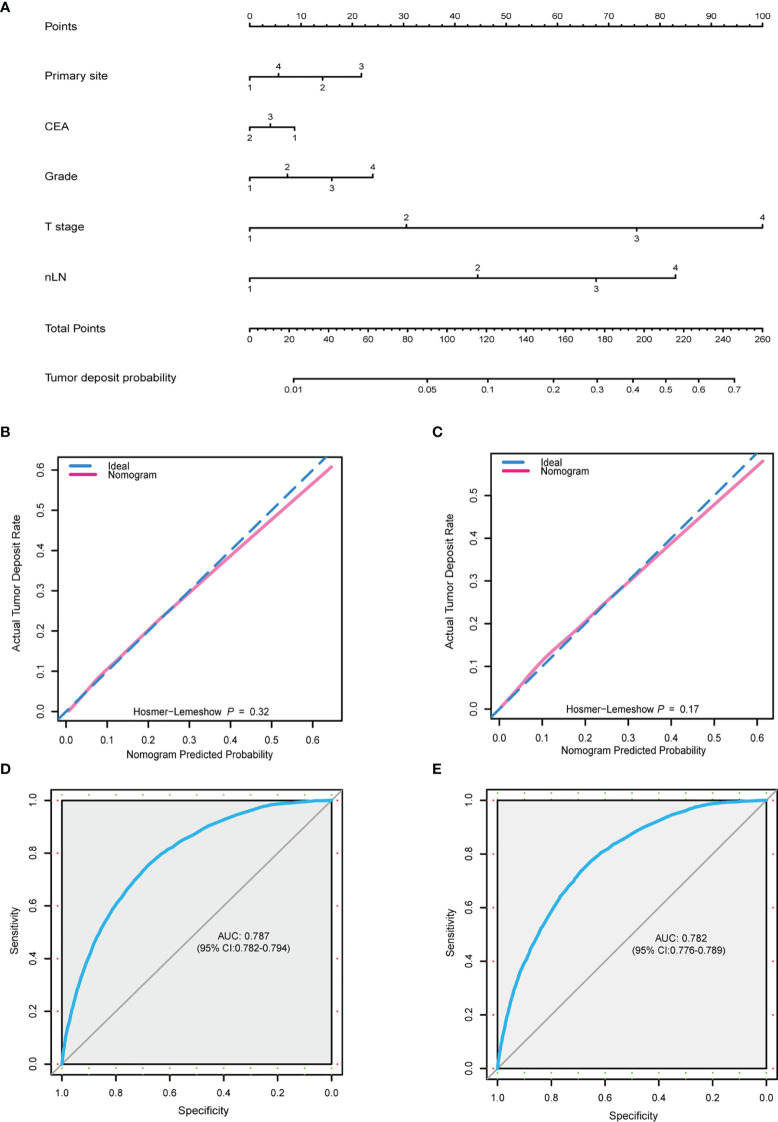
Nomogram for TD-positive prediction. **(A)** A predictive nomogram was built using multivariate logistic model. Primary site: 1, right colon; 2, left colon; 3, rectum; 4, overlapping/not otherwise specified. CEA: 1, positive; 2, negative; 3, unknown. Grade: 1, well; 2, moderately; 3, poorly; 4, undifferentiated. T stage: 1, T1; 2, T2; 3, T3; 4, T4. nLN: 1, 0; 2, 1–3; 3, 4–6; 4, >7. **(B, C)** Calibration plot of the predictive nomogram from the training **(B)** and validation **(C)** set. **(D, E)** ROC curve of the nomogram from the training set (AUC = 0.787, 95% CI: 0.782–0.794) and validation set (AUC = 0.782, 95% CI: 0.776–0.789). TD, tumor deposit; CEA, carcinoembryonic antigen; nLN, number of positive lymph nodes; ROC, receiver operating characteristic; AUC, area under the receiver operating characteristic curve.

In order to comprehensively predict TD presence probability, a nomogram was built based on the results of the multivariate logistic regression model including primary site, grade, T stage, nLN, and CEA (**Figure 2A**). The C-index of this predictive model was 0.785 (95% CI: 0.781–0.790). Beta-coefficients of the multivariate logistic regression model were used for the assignment of the score. By adding up all scales, the probability of TD presence in patients with CRC-NDM was predictable. In this model, the T stage and nLN were the largest contributors to the prediction of TD presence. Corresponding score assignments for every variable in this model are shown in **Supplementary Table 1**. Internal validation was carried out to examine the performance of this nomogram model. The calibration plots (Hosmer–Lemeshow test, *p* = 0.32 and *p* = 0.17, respectively) (**Figures 2B, C**) demonstrated that nomogram prediction was highly consistent with actual observations in both the training and validation data sets. The AUC for the training set was 0.787 (95% CI: 0.782–0.794) (**Figure 2D**) and 0.782 (95% CI: 0.776–0.789) for the validation set (**Figure 2E**). The baseline clinical characteristics of the training set and the validation set are shown in **Supplementary Table 2**.

### Univariate and Multivariate Analyses for Colorectal Cancer-Specific Death in Postoperative Colorectal Cancer With No Distant Metastasis Patients With Tumor Deposit Presence Using Competing-Risk Regression Model

**Figure 3:**
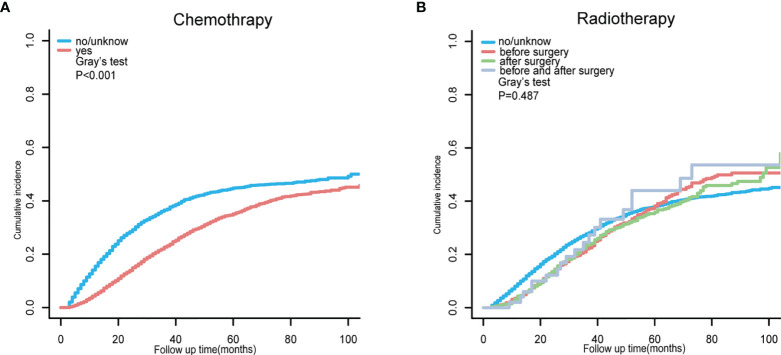
Cumulative incidence function curve for CCSD in postoperative CRC-NDM patients with TD, stratified by chemotherapy **(A)** and radiotherapy **(B)**. CCSD, colorectal cancer-specific death; CRC-NDM, colorectal cancer with no distant metastasis; TD, tumor deposit.

After exploring the clinical risk factors of TD presence in patients with CRC-NDM, we then used a competing-risk regression model to analyze the CCSD for postoperative CRC-NDM patients with TD. As presented in **Supplementary Table 3**, the univariate model indicated that age, marital status, primary site, histology, grade, tumor size, T stage, nLN, CEA, and chemotherapy were all significantly associated with CCSD in postoperative CRC-NDM patients with TD (Gray’s test, *p* < 0.05). Chemotherapy can significantly reduce CCSD (**Figure 3A**) (Gray’s test, *p* < 0.001). Radiotherapy before surgery, radiotherapy after surgery, and radiotherapy both before and after surgery all failed to reduce CCSD (**Figure 3B**) (Gray’s test, *p* = 0.487).

**Figure 4:**
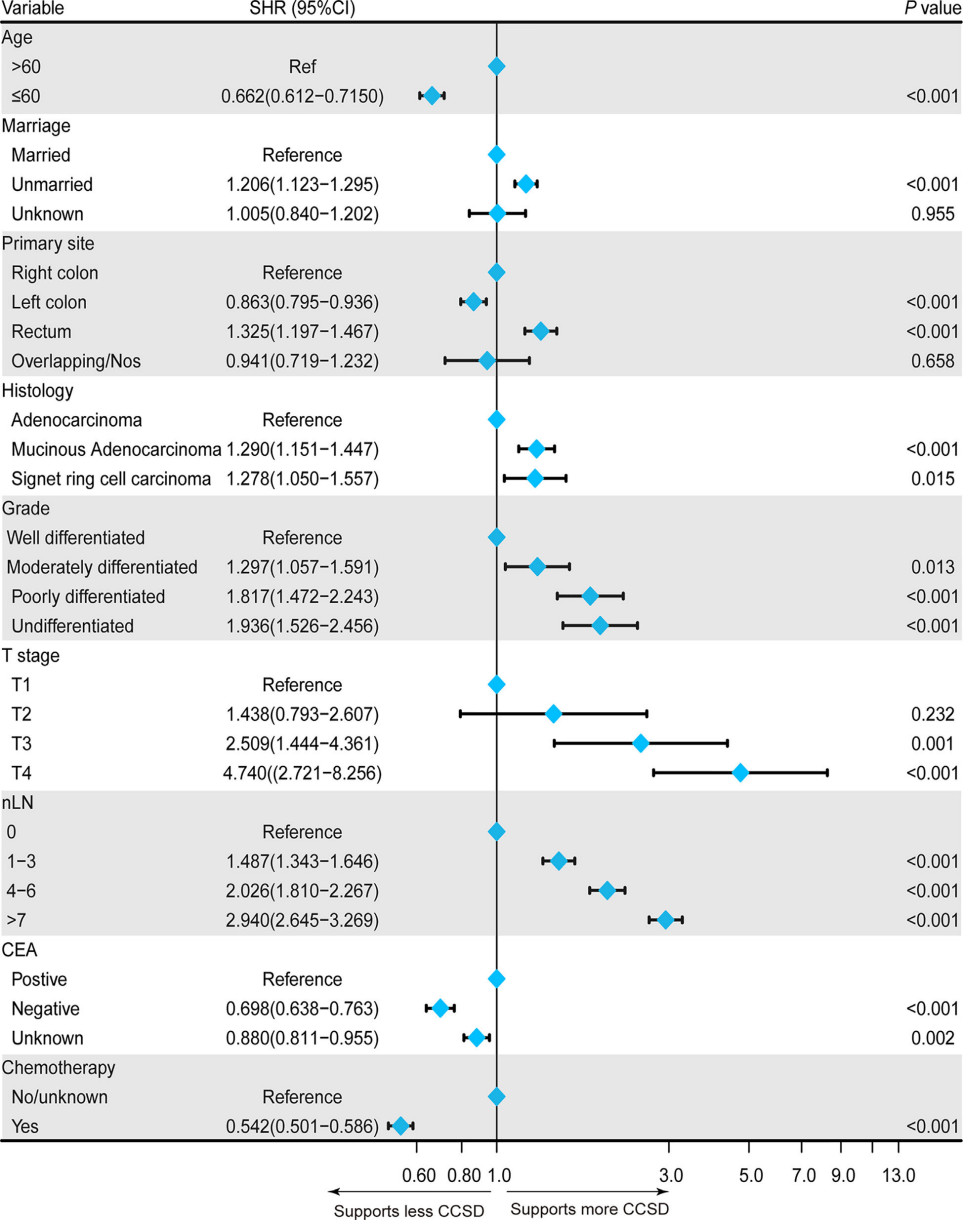
Forest plot showing the independent prognostic factors for the colorectal cancer-specific death (CCSD) in postoperative CRC-NDM patients with TD presence.

Then, all statistically potential independent factors (age, marital status, primary site, histology, grade, tumor size, T stage, nLN, CEA, and chemotherapy) selected from the univariate model were further incorporated in the multivariate competing-risk regression model. The result of multivariate model further confirmed that age ≤ 60 years (SHR = 0.662, 95% CI: 0.612–0.715), CEA-negative (SHR = 0.698, 95% CI: 0.638–0.763), the primary site of the left colon (SHR = 0.863, 95% CI: 0.795–0.936), and chemotherapy (SHR = 0.542, 95% CI: 0.501–0.586) were independently associated with less CCSD. Being unmarried (SHR = 1.206, 95% CI: 1.123–1.295), the primary site of rectum (SHR = 1.325, 95% CI: 1.197–1.467), the histology of mucinous adenocarcinoma (SHR = 1.290, 95% CI: 1.151–1.447), or signet ring cell carcinoma (SHR = 1.278, 95% CI: 1.050–1.557) were independently associated with higher CCSD. In addition, poorer grades, more nLN, and higher T stage (except for T2) were also independently associated with higher CCSD. The detailed results of multivariate competing-risk regression are presented in **Figure 4**.

### Construction and Validation of a Prognostic Nomogram for Predicting Colorectal Cancer-Specific Death in Postoperative Colorectal Cancer With No Distant Metastasis Patients With Tumor Deposit Presence

**Figure 5:**
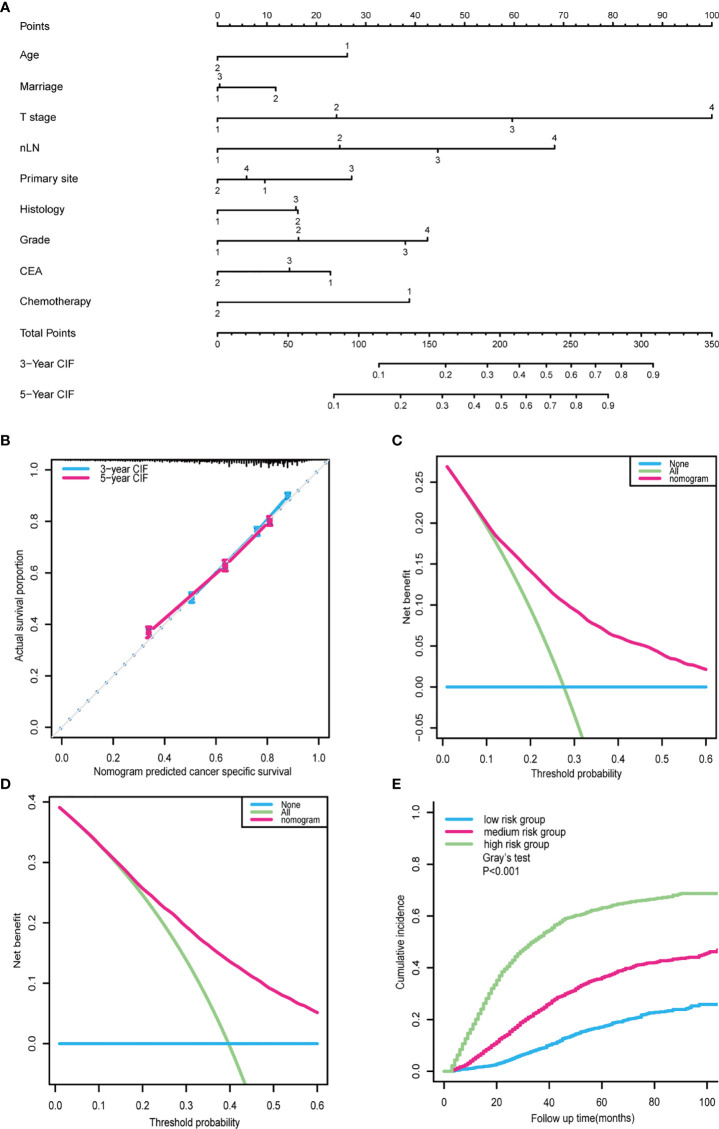
Nomogram for outcome prediction in postoperative CRC-NDM patients with TD. **(A)** A prognostic nomogram was built based on a multivariate competing-risk model. Age: 1, >60; 2, ≤60. Marital status: 1, married; 2, unmarried; 3, unknown. T stage: 1, T1; 2, T2; 3, T3; 4, T4. nLN: 1, 0; 2, 1–3; 3, 4–6; 4, >7. Primary site: 1, right colon; 2, left colon; 3, rectum; 4, overlapping/not otherwise specified. Histology: 1, adenocarcinoma; 2, mucinous adenocarcinoma. Grade: 1, well; 2, moderately; 3, poorly; 4, undifferentiated. CEA: 1, positive; 2, negative; 3, unknown. Chemotherapy: 1, no/unknown; 2, yes. **(B)** Calibration curve for predicting 3- and 5-year CCSD. **(C)** Decision curves for predicting 3-year CCSD nomogram. **(D)** Decision curves for predicting 5-year CCSD nomogram. **(E)** The cohort was classified into high-, medium-, and low-risk subgroups based on the prognostic nomogram. CRC-NDM, colorectal cancer with no distant metastasis; TD, tumor deposit; CEA, carcinoembryonic antigen; CCSD, colorectal cancer-specific death.

A prognostic nomogram was constructed using a multivariate competing-risk regression model including independent prognostic factors associated with CCSD for postoperative CRC-NDM patients with TD presence (**Figure 5A**). Age, marital status, primary site, histology, grade, T stage, nLN, CEA, and chemotherapy were incorporated into the model. By adding up all scales, we can give estimates of 3- and 5-year CCSD for each specific patient. Score assignment is shown in **Supplementary Table 4**. This prognostic model displayed acceptable accuracy in predicting CCSD, with a C-index of 0.727 (95% CI = 0.717–0.737). The calibration plots showed good consistency between the model prediction and actual observations for 3- and 5-year CCSD (**Figure 5B**). As displayed in **Figures 5C, D**, the DCA curves further confirmed the net benefit of our prognostic models in a wide range of threshold probabilities.

Taking a further step, the prognostic score (PS) of each patient was calculated by summing up scores of all variables according to this prognostic nomogram. Using the 25% and 75% of PS as the cutoff, all patients can be divided into three subgroups: low CCSD-PS, 0–142; medium CCSD-PS, 143–208; and high CCSD-PS, >208. Then, CCSD-PS was confirmed to be a strong prognostic factor to differentiate the whole cohort (**Figure 5E**). Specifically, the 5-year of CCSD was 17.16% in the low-risk subgroup, 36.20% in the medium-risk subgroup, and 63.19% in the high-risk subgroup (*p* < 0.001). Then, we performed a subgroup analysis stratified by the TNM stage to validate the efficacy of the prognostic nomogram (**Supplementary Figure 1**).

## Discussion

With the development of CRC screening and endoscopic technology, more CRC patients can be diagnosed and treated at an early stage. However, there are still about 80% of patients who had already developed to an advanced stage at the diagnosis of CRC, and about half of them had DM such as in the lung and liver (21). TD in CRC has gained extensive attention for many years. Recently, increasing research reported that TD was a kind of dissemination of primary tumor, associated with a poor prognosis (22). It is worth mentioning that our research firstly clarified the independent clinical risk factors for the presence of TD in patients with CRC-NDM and the prognostic factors for postoperative CRC-NDM patients with TD.

Nomograms are handy and effective predictive tools that can assist users in effectively predicting an event. For clinical application, nomograms have been used to predict the efficacy of adjuvant chemotherapy, survival rate, and recurrence in CRC (23–25). In our study, a nomogram was established for predicting the risk of TD presence in patients with CRC-NDM incorporating significant clinicopathological characteristics selected from multivariate logistic regression. Calibration plots displayed that nomogram prediction was highly consistent with the actual observation. The AUC of the receiver operating characteristic (ROC) curve for both training and validation sets >0.7 suggested that this predictive model had a relatively acceptable accuracy and good discrimination.

Of note, TD is not equal to LNM. Some clinical studies have reported that valuable information will be lost when we allocate TD as N1c and only consider TD in CRC patients without LNM (16). Therefore, it is recommended to add the number of TD when counting LNM, so as to improve the prognostic accuracy of TNM staging (14). However, other studies have shown that TD should be reported differently from LNM because patients with TD have a worse prognosis (22). What is more, several research has reported the biological difference between TD and LNM. For example, KRAS mutation and Twist upregulation were strongly associated with TD presence, while Snail overexpression was significantly correlated with LNM (26, 27). The origin of TD and LNM were also different. Several investigators reviewed the serial sectioning of TD samples, and all of them reported that the presence of TD was associated with a higher perineural, lymphatic, and vascular invasion rate (28). In addition, vascular and perineural invasion were more common among TD-positive patients, which may partially explain the worse prognosis than LNM (16, 29).

Through a multivariate logistic regression model, we discovered that primary site, CEA, T stage, grade, and nLN were independent predictive factors for the presence of TD in patients with CRC-NDM. In addition, both the T stage and nLN were powerful contributors to the prediction. A higher T stage and more nLN had a higher probability of TD. One possible reason is that a deep infiltration and increased nLN suggest perineural, lymphatic, and vascular involvement. For the risk factors of LNM in CRC, a recent meta-analysis summarized the independent variables associated with LNM in early-stage CRC and reported that depth of tumor invasion, rectal location of the tumor, and higher differentiation grade were significantly associated with LNM (30). A previous study showed that left colon cancer has a higher rate of LNM than right colon cancer, as well as a lymphatic invasion (31). In the present study, the primary site of the left colon was also associated with a higher probability of TD than the right colon. The difference in TD presence can also be explained by the different lymphatic vessel involvement rates between right and left colon cancer. Moderately differentiated, poorly differentiated, and undifferentiated CRC lesions had a higher probability of TD than well-differentiated CRC lesions. CEA was used for follow-up and recurrence monitoring after therapy for many years, and a lot of previous studies reported that CRC patients with continuous CEA-positive tended to have worse survival (32). In this study, we found that CRC-NDM patients with CEA-negative have a lower risk of TD presence. For the histology of CRC, signet ring cell carcinoma, mucinous adenocarcinoma, and adenocarcinoma have a comparable TD-positive risk in patients with CRC-NDM.

For the survival analysis of postoperative CRC-NDM patients with TD presence, we aim to identify prognostic factors for these patients after surgery and build a prognostic nomogram to help select patients with worse outcomes. For the survival framework, the traditional Kaplan–Meier method and Cox proportional hazards regression model may overestimate the risk of cancer-specific death when competing events exist (e.g., cardiovascular and cerebrovascular accidents, and treatment-related deaths) (33). However, the competing-risk model used in our avoided this limit and might be more reliable.

In agreement with other studies for the CRC survival analysis, our survival analysis for patients with TD also uncovered that CEA positive, poorer grade, more nLN, and higher T stage (except for T2) were independently associated with worse prognosis (32, 34). Marital status was found to be an independent risk factor for CCSD in our study, and being unmarried had a higher CCSD. Social support is an important part of the management of cancer patients. A previous study by Aizer et al. indicated that unmarried patients are at a higher risk of CCSD than married patients, probably due to the fact that unmarried CRC patients were often diagnosed at an advanced stage and were less likely to receive timely treatment than married patients (35). For the primary site, compared with right colon cancer, rectal cancer showed higher CCSD, while the left colon cancer was associated with less CCSD. The possible explanation is that the different intrinsic biological behaviors of the right and left colon cancer and the higher rate of BRAF mutant cases in right colon cancer are related to a more aggressive clinical behavior (36). A previous study by Hashiguchi et al. stated that patients with rectal cancer reportedly experienced DM and local recurrence more frequently than those with colon cancer (37). So rectal cancer showed that higher CCSD in our study could be also explained. For the histology of CRC, a previous study revealed that adenocarcinoma and mucinous adenocarcinoma have a similar prognosis, while signet ring cell carcinoma tends to have a worse prognosis (38). In our analysis for postoperative CRC-NDM patients with TD, histology of signet ring cell carcinoma and mucinous adenocarcinoma were significantly associated with a worse prognosis than adenocarcinoma. The effect of chemotherapy on CRC patients with TD was inconsistent in previous studies. Shi et al. found that the presence of TD and its number did not affect the benefit of chemotherapy in stage III CRC (39), while Li et al. reported that patients with TD did not display a disease-free survival (DFS) benefit from chemotherapy (40). However, in our study, for the postoperative CRC-NDM patients with TD, chemotherapy (SHR = 0.542, 95% CI: 0.501–0.586) was independently associated with a favorable prognosis. As mentioned above, TD-positive patients tend to invade blood vessels and metastasize to distant organs, and systemic chemotherapy may reduce the dissemination of tumor cells, resulting in prolonged survival time. A prognostic nomogram was also constructed to help clinicians identify poor-outcome individuals in postoperative CRC-NDM patients with TD presence, and the 5-year of CCSD was associated with the risk degree.

However, our study has some deficits. Firstly, this study was a large-scale retrospective study, and there still remain miscoding and selection biases. Secondly, our prognostic nomograms demonstrated good performance for risk stratification in the internal validation, but further external validation is needed to determine whether the nomogram can be applied to a wider population. Thirdly, in our validation of prognostic nomogram stratified by TNM stage, we only have 82 stage I patients with TD presence, including 77 low-risk groups, 5 medium-risk groups, and 0 high-risk groups. Fine and Gray’s test showed that there was no significance between the low-risk group and medium-risk group in stage I patients. In the future, we need more TD-positive patients to evaluate the efficacy of our prognostic nomogram in stage I patients. Fourthly, some meaningful variables may also be potential independent factors for the TD presence, such as molecular biomarkers (e.g., KRAS mutation, NRAS mutation, BRAF mutation, HER2 status, and microsatellite instability (MSI)). However, these variables are unavailable in the SEER database and could not be included in the present study. Incorporating these variables may further enhance the accuracy of the predictive model. Moreover, for the survival analysis, some variables such as Charlson Comorbidity Index (CCI), and circumferential resection margin (CRM) also cannot be adjusted in our prognostic model due to the limitation of the SEER database. Finally, because the SEER database lacked detailed information on the chemotherapy or radiotherapy, we were unable to estimate the cumulative incidence of CCSD according to the regimen of chemotherapy or radiotherapy. We also could not definitively differentiate patients who did not receive chemotherapy from patients with unknown information of chemotherapy or radiotherapy.

In conclusion, we constructed an easily predictive nomogram in identifying the high-risk TD-positive patients, which may remind surgeons and pathologists to carefully observe surgical field and resection specimens to find the harboring TD lesion. A prognostic nomogram was built to help clinicians to identify those individuals with poor outcomes. For the high-risk or medium-risk subgroup, additional chemotherapy and close follow-up may bring benefits for the TD-positive patients rather than radiotherapy.

## Data Availability Statement

The original contributions presented in the study are included in the article/**Supplementary Material**. Further inquiries can be directed to the corresponding authors.

## Ethics Statement

The studies involving human participants were reviewed and approved by the Ethics Committee of the Second Affiliated Hospital of Zhejiang University School of Medicine. Written informed consent for participation was not required for this study in accordance with the national legislation and the institutional requirements.

## Author Contributions

MX and LS designed the study. JN and JS contributed to the conception of the study. JC, ZZ, and WR contributed significantly to the analysis and manuscript preparation. JC and YS performed the data analyses and wrote the manuscript. YS and WR helped perform the analysis with constructive discussions. All authors contributed to the article and approved the submitted version.

## Funding

This work was financially supported by the National Foundation of Natural Science of China (No. 82073229).

## Conflict of Interest

The authors declare that the research was conducted in the absence of any commercial or financial relationships that could be construed as a potential conflict of interest.

## Publisher’s Note

All claims expressed in this article are solely those of the authors and do not necessarily represent those of their affiliated organizations, or those of the publisher, the editors and the reviewers. Any product that may be evaluated in this article, or claim that may be made by its manufacturer, is not guaranteed or endorsed by the publisher.
