# Supplementary figures and images for: Predictive and Prognostic Assessment Models for Tumor Deposit in Colorectal Cancer Patients With No Distant Metastasis
Source: Front Oncol. 2022 Feb 16;12:809277. doi: 10.3389/fonc.2022.809277 (PMC8888919; doi:10.3389/fonc.2022.809277)

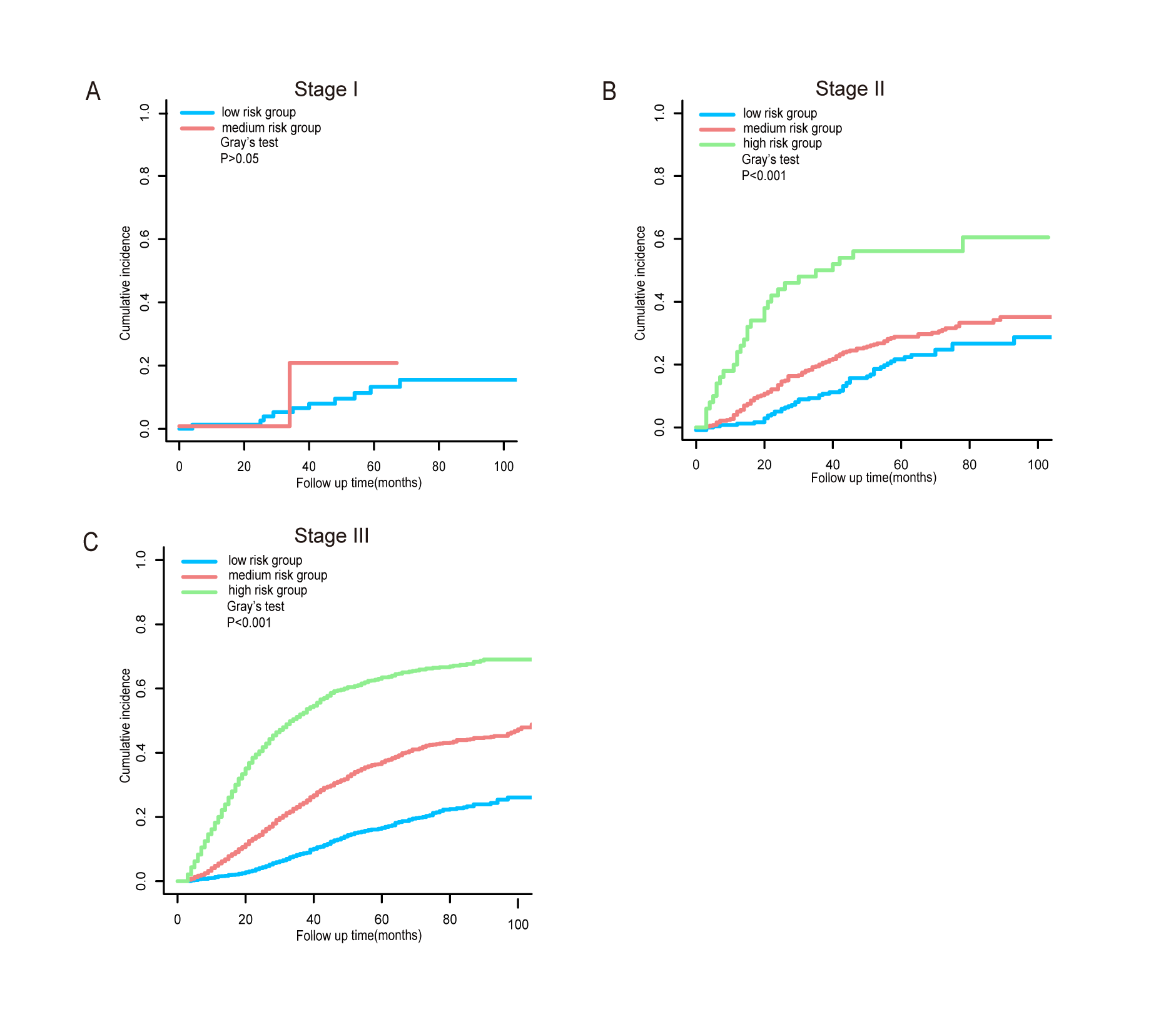

Supplement: Supplementary file 3 [file Image_1.tif]
