# Supplementary material for: Predictive and Prognostic Assessment Models for Tumor Deposit in Colorectal Cancer Patients With No Distant Metastasis
Source: Front Oncol. 2022 Feb 16;12:809277. doi: 10.3389/fonc.2022.809277 (PMC8888919; doi:10.3389/fonc.2022.809277)
Supplement: Supplementary file 4 [file Table_1.pdf]

Supplementary Table 1 Point assignment of nomogram risk scores for TD probability

Variable	Number	Score
Primary site		
Right colon	1	0
Left colon	2	14
Rectum	3	22
Overlapping/Nos	4	6
Grade		
Well differentiated	1	0
Moderately differentiated	2	7
Poorly differentiated	3	16
Undifferentiated	4	24
T stage		
T1	1	0
T2	2	31
T3	3	75
T4	4	100
nLN		
0	1	0
1-3	2	44
4-6	3	68
>7	4	83
CEA		
Postive	1	9
Negative	2	0
Unknown	3	4
