# Supplementary material for: Predictive and Prognostic Assessment Models for Tumor Deposit in Colorectal Cancer Patients With No Distant Metastasis
Source: Front Oncol. 2022 Feb 16;12:809277. doi: 10.3389/fonc.2022.809277 (PMC8888919; doi:10.3389/fonc.2022.809277)
Supplement: Supplementary file 5 [file Table_2.pdf]

Supplementary Table 2 Baseline clinical characteristics of the training set and the validation set for the predictive nomogram construction.

Variable	Training set (n=60243)				Validation set (n=40531)			
	TD-negative (n=53801)	%	TD-positive (n=6442)	%	TD-negative (n=36238)	%	TD-positive (n=4293)	%
Age at diagnosis								
≤60	19102	35.50	3891	60.40	12805	35.34	2597	60.49
>60	34699	64.50	2551	39.60	23433	64.66	1696	39.51
Gender								
Female	26446	49.16	3129	48.57	17911	49.43	2065	48.10
Male	27355	50.84	3313	51.43	18327	50.57	2228	51.90
Race								
White	42309	78.64	5021	77.94	28570	78.84	3307	77.03
Black	6085	11.31	703	10.91	4064	11.21	492	11.46
Other	5407	10.05	718	11.15	3604	9.95	494	11.51
Primary site								
Right colon	27062	50.30	2829	43.91	18238	50.33	1833	42.70
Left colon	18078	33.60	2451	38.05	12161	33.56	1682	39.18
Rectum	7930	14.74	1051	16.31	5361	14.79	710	16.54
Overlapping/Nos	731	1.36	111	1.72	478	1.32	68	1.58
Histology				0.00				0.00
Adenocarcinoma	49502	92.01	5766	89.51	33339	92.00	3854	89.77
Mucinous Adenocarcinoma	3980	7.40	532	8.26	2669	7.37	359	8.36
Signet ring cell carcinoma	319	0.59	144	2.24	230	0.63	80	1.86
Grade								
Well differentiated	4614	8.58	265	4.11	3089	8.52	206	4.80
Moderately differentiated	40871	75.97	4302	66.78	27476	75.82	2910	67.78
Poorly differentiated	6940	12.90	1507	23.39	4758	13.13	941	21.92
Undifferentiated	1376	2.56	368	5.71	915	2.52	236	5.50
Tumor size								
≤2cm	8464	15.73	379	5.88	5797	16.00	262	6.10
≤3cm	8388	15.59	895	13.89	5711	15.76	563	13.11
≤5cm	19192	35.67	2514	39.03	12757	35.20	1722	40.11
>5cm	17757	33.00	2654	41.20	11973	33.04	1746	40.67
T stage								
T1	6862	12.75	88	1.37	4499	12.42	49	1.14
T2	9779	18.18	289	4.49	6850	18.90	215	5.01
T3	30510	56.71	4055	62.95	20467	56.48	2727	63.52
T4	6650	12.36	2010	31.20	4422	12.20	1302	30.33
nLN								
0	36419	67.69	1752	27.20	24677	68.10	1230	28.65
0-3	12080	22.45	2220	34.46	7994	22.06	1459	33.99
3-6	3149	5.85	1156	17.94	2062	5.69	745	17.35
>7	2153	4.00	1314	20.40	1505	4.15	859	20.01
CEA								
Postive	11711	21.77	2026	31.45	7720	21.30	1332	31.03
Negative	21397	39.77	2143	33.27	14530	40.10	1370	31.91
Unknown	20693	38.46	2273	35.28	13988	38.60	1591	37.06
