# Supplementary material for: Predictive and Prognostic Assessment Models for Tumor Deposit in Colorectal Cancer Patients With No Distant Metastasis
Source: Front Oncol. 2022 Feb 16;12:809277. doi: 10.3389/fonc.2022.809277 (PMC8888919; doi:10.3389/fonc.2022.809277)
Supplement: Supplementary file 6 [file Table_3.pdf]

Supplementary Table 3 Univariate and multivariate competing risk regression analysis for colorectal cancer specific death (CCSD) in postoperative CRC-NDM patients with TD-positive

Variable	Univariate analysis		Multivariate analysis	
	SHR (95%CI)	*P* value	SHR (95%CI)	*P* value
Age				
>60	Ref		Ref	
≤60	0.636(0.592-0.683)	**<0.001**	0.662(0.612-0.7150)	**<0.001**
Gender			*NI*	
Female	Ref			
Male	0.975(0.910-1.044)	0.471		
Race			*NI*	
White	Ref			
Black	0.981(0.880-1.094)	0.731		
Other	0.963(0.864-1.074)	0.500		
Marriage				
Married	Ref		Ref	
Unmarried	1.315(1.226-1.410)	**<0.001**	1.206(1.123-1.295)	**<0.001**
Unknown	1.044(0.872-1.249)	0.64	1.005(0.840-1.202)	0.955
Primary site				
Right colon	Ref		Ref	
Left colon	0.676(0.625-0.731)	**<0.001**	0.863(0.795-0.936)	**<0.001**
Rectum	0.795(0.724-0.872)	**<0.001**	1.325(1.197-1.467)	**<0.001**
Overlapping/Nos	1.009(0.767-1.329)	0.07	0.941(0.719-1.232)	0.658
Histology				
Adenocarcinoma	Ref		Ref	
Mucinous Adenocarcinoma	1.458(1.299-1.636)	**<0.001**	1.290(1.151-1.447)	**<0.001**
Signet ring cell carcinoma	2.465(2.016-3.015)	**<0.001**	1.278(1.050-1.557)	0.015
Grade				
Well differentiated	Ref		Ref	
Moderately differentiated	1.278(1.044-1.563)	**0.017**	1.297(1.057-1.591)	0.013
Poorly differentiated	2.343(1.905-2.882)	**<0.001**	1.817(1.472-2.243)	**<0.001**
Undifferentiated	2.772(2.188-3.513)	**<0.001**	1.936(1.526-2.456)	**<0.001**
Tumor size				
≤2cm	Ref		Ref	
≤3ccm	1.430(1.192-1.716)	**<0.001**	1.175(0.971-1.422)	0.097
≤5cm	1.427(1.208-1.686)	**<0.001**	1.038(0.871-1.238)	0.675
>5cm	1.824(1.546-2.152)	**<0.001**	1.103(0.925-1.315)	0.276
T stage				
T1	Ref		Ref	
T2	1.556(0.863-2.808)	0.142	1.438(0.793-2.607)	0.232
T3	3.355(1.947-5.781)	**<0.001**	2.509(1.444-4.361)	**0.001**
T4	7.057(4.092-12.168)	**<0.001**	4,740((2.721-8.256)	**<0.001**

Supplementary Table 3 (Contiuned)

Variable	Univariate analysis		Multivariate analysis	
	SHR (95%CI)	*P* value	SHR (95%CI)	*P* value
nLN				
0	Ref		Ref	
1-3	1.365(1.236-1.507)	**<0.001**	1.487(1.343-1.646)	**<0.001**
4-6	1.937(1.738-2.160)	**<0.001**	2.026(1.810-2.267)	**<0.001**
>7	3.002(2.717-3.318)	**<0.001**	2.940(2.645-3.269)	**<0.001**
CEA				
Positive	Ref		Ref	
Negative	0.635(0.582-0.693)	**<0.001**	0.698(0.638-0.763)	**<0.001**
Unknown	0.890(0.821-0.965)	0.005	0.880(0.811-0.955)	0.002
Chemotherapy				
No/unknown	Ref		Ref	
Yes	0.592(0.551-0.636)	**<0.001**	0.542(0.501-0.586)	**<0.001**
Radiation				
No/unknown	Ref		*NI*	
Before surgery	0.944(0.850-1.050)	0.293		
After surgery	0.924(0.814-1.050)	0.225		
Before and after surgery	1.082(0.714-1.638)	0.71		

Note: ≤3 cm, 2 cm < tumor size ≤ 3 cm; ≤5 cm, 3 cm < tumor size ≤ 5 cm.

Abbreviations: SHR, subdistribution hazard ratio; Nos, not otherwise specified; TD, tumor deposit; nLN, number of positive lymph node; CEA, carcinoma embryonic antigen; NI, not included; Ref, reference; CI, confidence interval.
