# Supplementary material for: Predictive and Prognostic Assessment Models for Tumor Deposit in Colorectal Cancer Patients With No Distant Metastasis
Source: Front Oncol. 2022 Feb 16;12:809277. doi: 10.3389/fonc.2022.809277 (PMC8888919; doi:10.3389/fonc.2022.809277)
Supplement: Supplementary file 7 [file Table_4.pdf]

Supplementary Table 4 Point assignment of nomogram risk scores for CCSD in postoperative CRC-NDM patients with TD-positive

Variable	Number	Score
Age		
>60	1	26
≤60	2	0
Marriage		
Married	1	0
Unmarried	2	12
Unknown	3	0
T stage		
T1	1	0
T2	2	24
T3	3	60
T4	4	100
nLN		
0	1	0
1-3	2	25
4-6	3	45
>7	4	68
Primary site		
Right colon	1	10
Left colon	2	0
Rectum	3	27
Overlapping/Nos	4	6
Histology		
Adenocarcinoma	1	0
Mucinous Adenocarcinoma	2	16
Signet ring cell carcinoma	3	16
Grade		
Well differentiated	1	0
Moderately differentiated	2	16
Poorly differentiated	3	38
Undifferentiated	4	42
CEA		
Postive	1	23
Negative	2	0
Unknown	3	15
Chemotherapy		
No/unknown	1	39
Yes	2	0
